# OTUB1 contributes to the stability and function of Influenza A virus NS2

**DOI:** 10.1371/journal.ppat.1012279

**Published:** 2024-05-30

**Authors:** Yu-Jyun Li, Chi-Yuan Chen, Yu-Shen Kuo, Yi-Wen Huang, Rei-Lin Kuo, Li-Kwan Chang, Jeng-How Yang, Chih-Ho Lai, Shin-Ru Shih, Ya-Fang Chiu

**Affiliations:** 1 Department of Microbiology and Immunology, Chang Gung University, Taoyuan, Taiwan; 2 Graduate Institute of Biomedical Sciences, Chang Gung University, Taoyuan, Taiwan; 3 Research Center for Emerging Viral Infections, Chang Gung University, Taoyuan, Taiwan; 4 Department of Biochemical Science and Technology, College of Life Science, National Taiwan University, Taipei, Taiwan; 5 Division of Infectious Diseases, Department of Medicine, Chang Gung Memorial Hospital, New Taipei, Taiwan; 6 Department of Laboratory Medicine, Linkou Chang Gung Memorial Hospital, Taoyuan, Taiwan; The Ohio State University, UNITED STATES

## Abstract

The influenza A virus (IAV) consists of 8 single-stranded, negative-sense viral RNA (vRNA) segments. After infection, vRNA is transcribed, replicated, and wrapped by viral nucleoprotein (NP) to form viral ribonucleoprotein (vRNP). The transcription, replication, and nuclear export of the viral genome are regulated by the IAV protein, NS2, which is translated from spliced mRNA transcribed from viral NS vRNA. This splicing is inefficient, explaining why NS2 is present in low abundance after IAV infection. The levels of NS2 and its subsequent accumulation are thought to influence viral RNA replication and vRNP nuclear export. Here we show that NS2 is ubiquitinated at the K64 and K88 residues by K48-linked and K63-linked polyubiquitin (polyUb) chains, leading to the degradation of NS2 by the proteasome. Additionally, we show that a host deubiquitinase, OTUB1, can remove polyUb chains conjugated to NS2, thereby stabilizing NS2. Accordingly, knock down of OTUB1 by siRNA reduces the nuclear export of vRNP, and reduces the overall production of IAV. These results collectively demonstrate that the levels of NS2 in IAV-infected cells are regulated by a ubiquitination-deubiquitination system involving OTUB1 that is necessary for optimal IAV replication.

## Author summary

The influenza A virus (IAV) protein, NS2, plays a critical role in virus replication, as the abundance of NS2 influences viral transcription, viral RNA (vRNA) synthesis, and viral ribonucleoprotein (vRNP) export from the nucleus. Interestingly, high levels of NS2 are known to inhibit IAV transcription yet promote vRNA synthesis. In this study, we found that ubiquitination destabilizes NS2, and a host deubiquitinase, OTUB1, was shown to deubiquitinate and stabilize NS2, thus contributing to NS2 accumulation. OTUB1 has previously been reported to be a negative regulator of host cellular antiviral defense, and its upregulation enhances virion production. Our study sheds additional light on the role of OTUB1 during IAV infection which can now be considered for the development of anti-influenza strategies.

## Introduction

The influenza A virus (IAV) genome is made up of 8 negative-sense, single-stranded viral RNA (vRNA) segments [1]. After infection and viral replication, synthesized vRNA is wrapped by viral nucleoprotein (NP) to form viral ribonucleoprotein (vRNP) [2], which is bound at the termini by RNA-dependent RNA polymerase (RdRp) [3,4]. Ultimately, vRNP is exported from the nucleus and packaged into virions for release from the host cell [4–6].

Importantly, although RdRp is required for viral genome transcription and viral RNA replication, these processes do not occur concurrently [7,8]. Rather, transcription of viral mRNA precedes complementary RNA (cRNA) and vRNA replication [7,8]. The timing of RNA synthesis is crucial to IAV maturation, and disruption of this process significantly impacts IAV propagation [9]. Earlier studies showed that IAV proteins are usually expressed immediately after infection [10,11]. However, NS2 is initially expressed at a low level after infection but accumulates over time to a high level when the virus reaches the late stage of infection [9]. Earlier studies that employed a transfection system to overexpress NS2 demonstrated that NS2 expression promotes cRNA and vRNA syntheses [12]. Based on these results, it is thought that accumulation of NS2 after IAV infection is critical to cRNA and vRNA replication [9]. Therefore, it is thought that NS2 serves as a molecular timer that triggers viral RNA replication after its accumulation, and is also involved in the temporal-spatial regulation of viral RNA synthesis events [9,12,13]. It is also known that NS mRNA, which is transcribed from the NS segment of IAV vRNA, encodes the NS1 protein. The mRNA also undergoes splicing to form NS2 mRNA, which is translated to the NS2 protein [14,15]. However, a weak 5’ splice site in NS mRNA causes inefficient splicing [16,17], explaining why NS2 is present in low abundance at the initial stage of IAV infection. In addition to promoting viral RNA replication, NS2 also serves as an adaptor that binds to vRNP, viral M1 protein, and interacts with the cellular CRM1 nuclear export machinery to facilitate vRNP nuclear export [18,19]. Alteration of the timing of NS2 expression and accumulation can influence the export of vRNP from the nucleus [9]. Moreover, NS2 has been reported to have other critical functions in viral propagation, such as facilitation of IAV particle budding from the host cell via interaction with a host ATPase [20]; participation in the regulation of host interferon-β (IFN-β)-mediated innate immunity [21,22]; and potential involvement in host adaptation [23–25]. Uncovering the mechanisms that affect NS2 accumulation and translocation are needed to understand IAV production in host cells.

Otubain 1 (OTUB1) is a member of the OTU domain family of deubiquitinases (DUBs) [26,27]. OTUB1 cleaves both K48-linked and K63-linked polyubiquitination (polyUb) chains to stabilize its target proteins [26,27]. An earlier study revealed that the D88 and C91 residues of OTUB1 are critical to its deubiquitinase (DUB) activity [26]. In addition to canonical DUB activity, OTUB1 can inhibit ubiquitination by interacting with ubiquitin charged E2 to prevent ubiquitin transfer to a target protein [28,29]. It has also been reported that OTUB1 promotes apoptosis by preventing the degradation of p53 via the MDM2/MDMX-mediated ubiquitination proteasome system [30,31]. Additionally, OTUB1 has been reported to play a role in IAV propagation and host antiviral defense. Previous research has shown that during viral infection, OTUB1 translocates from the nucleus to the mitochondrial membrane, where it activates RIG-I-dependent immune signaling; however, IAV NS1 can trigger proteasomal degradation of cellular OTUB1 to dampen this antiviral immune response [32]. Moreover, Li *et al*. [33] have shown that OTUB1 suppresses host innate immunity by repressing TRAF-3 and IRF3. These findings indicate that the location, as well as the abundance of OTUB1 in host cells influence influenza infection. Our study adds a piece to this puzzle by characterizing a role for OTUB1 which provides the rationale for considering development of anti-viral strategies aimed at the OTUB1 pathway.

## Materials and methods

### Cell lines, bacterial strains, and virus

HEK293T, A549, H1299, Vero-E6, and Madin-Darby canine kidney (MDCK) cells were cultured at 37°C in Dulbecco’s Modified Eagle’s Medium (DMEM; Hyclone), supplemented with 10% fetal bovine serum (FBS; Hyclone). *Escherichia coli* BL21(DE3) cells were cultured in LB medium [34]. Influenza A/Puerto Rico/8/1934 (H1N1) and A/Hong Kong/8/68 (H3N2) were amplified in 9-day-old chicken embryos and MDCK cells, respectively, and titered by plaque formation assay on a monolayer of MDCK cells. For determining viral growth characteristics, A549 or Vero-E6 cells were infected with viruses at a multiplicity of infection (MOI) of 0.01 or 2, and for the time indicated in the text and figure legends. The NS cDNA in a reverse genetic plasmid, pHW-NS [35], was mutated with a Q5 site-directed mutagenesis kit (NEB BioLabs) to introduce mutations in the NS2 gene of A/Puerto Rico/8/1934 (H1N1) strain with all lysine residues substituted by arginine except for K64 or K88 but without disturbing the amino acid sequence in NS1 to generate pHW-NS-NS2(K64) or pHW-NS-NS2(K88), respectively. Plasmids pHW-NS-NS2(K64) or pHW-NS-NS2(K88) along with the other 7 IAV reverse genetics plasmids (pHW-PB1, pHW-PB2, pHW-PA, pHW-NP, pHW-NA, pHW-M, and pHW-HA) was transfected into HEK293T cells to produce IAV[NS2(K64)] or IAV[NS2(K88)], respectively, and the virus was amplified in 9-day-old chicken embryos.

### Antibodies

The antibodies used in this study included anti-HA (#3724, Cell Signaling), anti-V5 (#13202, Cell Signaling; T0057, Affinity Biosciences), anti-Flag M2 (F3165; Sigma), anti-OTUB1 (GTX57636, GeneTex), anti-lamin-B1 (GTX103292, GeneTex), anti-α-tubulin (Clone DM1A, T9026; Sigma), anti-ubiquitin (Ub) (A11227, Abclonal), anti-PB2 (GTX125926, GeneTex), anti-NP (GTX125989, GeneTex), anti-M1 (GTX125928, GeneTex), anti-NS1 (GTX125990, GeneTex), anti-NA (GTX125974, GeneTex), anti-NS2 (GTX125953, GeneTex), anti-GST (sc-459, Santa Cruz Biotechnology), and anti-His (05–949, Millipore) antibodies.

### Plasmids

Plasmids pHW-PB1, pHW-PB2, pHW-PA, and pHW-NP were described elsewhere [35]. The NA cDNA was amplified with primers 5’-GCGCGTCTCAGGGAGCGAAAGCAGGAGTTTAAAATGAAT and 5’-GCGCGTCTCATATTAGTAGAAACAAGGAGTTTTTTGAACAG using pHW-NA as a template [35]. The cDNA was inserted into the *Bsm*BI site in pHH21 to generate a plasmid (pHH-NA) transcribing NA vRNA from a human RNA polymerase I (PolI) promoter and the mouse RNA polymerase I terminator [36]. A DNA fragment encoding a V5 tag [37] was synthesized and inserted into the *Nhe*I-*Bam*HI sites in pcDNA3.1(+) (Invitrogen) to generate pcDNA-NV5. NS2 cDNA amplified by RT-PCR was inserted into the *Eco*RI-*Eco*RV sites in pcDNA-HA, or *Eco*RV-*Xho*I sites in pET32a (Novagen), to create pHA-NS2 or pET-NS2, respectively. The ubiquitin gene, which was amplified using primers 5’-ATCGGATCCATGCAGATCTTCGTCAAGACG and 5’-TATGATATCTCAACCACCTCTTAGTCTTAAGAC, was inserted into the *Bam*HI-*Eco*RV sites in pcDNA-NV5 to generate pV5-Ub. The sequence in the Ub gene in pV5-Ub was mutated using the Q5 Site-Directed Mutagenesis Kit (NEB) to generate pV5-Ub(K0), which encodes a Ub mutant with all the 7 lysine residues substituted by arginine. Plasmid pFlag-OTUB1 was kindly provided by Cynthia Wolberger [29]. The OTUB1 gene was amplified using primers 5’-ATCGGATCCATGGCGGCGGAGGAAC and 5’-CGACTCGAGCTATTTGTAGAGGATATCGTAGTG; the gene was inserted into the *Bam*HI-*Xho*I sites in p3xFlag-myc-CMV26 (Sigma), pcDNA-NV5, pET-32a, or pGEX-4T1 (GE Healthcare) to yield pFlag-OTUB1, pV5-OTUB1, pET-OTUB1, or pGST-OTUB1, respectively. D88A and C91S substitutions were introduced into the OTUB1 gene in pFlag-OTUB1 by site-directed mutagenesis with a Q5 Site-Directed Mutagenesis Kit (NEB) using primers 5’-AACAGCTTCTATCGGGCTTTCGGATTC and 5’-GCCAGCAGGCCTGGTCTTGCGGAT to create pFlag-mOTUB1. Three stop-codons were introduced into the NS1 open reading frame in pHW-NS to yield pNS2. The plasmid does not express NS1, and only allows expression of NS2 after RNA splicing.

### siRNA knockdown

The expression of OTUB1 was knocked down by transfecting two double-stranded siRNAs against OTUB1 with the sequence of 5’-CCGACUACCUUGUGGUCUA and 5’-UGGAUGACAGCAAGGAGUU and two extra 2’-deoxythymidine (dT) at their 3’ terminus (Helix Tech). This study also transfected cells with scramble siRNA (5’-UUCUCCGAACGUGUCACGU) as a control. siRNAs (50 nM) were transfected into A549 or Vero-E6 cells using the TransIT-X2 Dynamic Delivery System (Mirus Bio).

### Coimmunoprecipitation

HEK293T lysates were prepared by sonication in phosphate-buffered saline (PBS) containing 0.1% Triton X-100 and 0.5% protease inhibitor cocktail (Calbiochem) according to a method described elsewhere [38]. Proteins in the lysate were immunoprecipitated (IP) with mouse anti-HA agarose beads (Sigma, A2095) or mouse anti-Flag M2 agarose beads (Sigma, A2220) for 2 h at 4°C. After immunoprecipitation, proteins bound to the beads were washed three times with PBS containing 0.1% Triton X-100, eluted with 30 μl 2× electrophoresis sample buffer, and detected by immunoblotting (IB) according to a method described elsewhere [38].

### Denaturing immunoprecipitation

HEK293T transfected transiently were treated with 5 μM MG132 for 14 h and washed twice with PBS containing 0.05% N-ethylmaleimide (NEM). Cells were then lysed at 95°C for 15 min with denaturing IP buffer, which contained a 1:3 ratio of buffer I (5% SDS, 150 mM Tris-HCl at pH 6.7, and 30% glycerol) to buffer II (0.1% SDS, 150 mM Tris-HCl at pH 8.2, 5 mM NaCl, 0.5% NP-40, and 0.1% sodium azide) [39–41]. Proteins in the cell lysate were immunoprecipitated with anti-HA agarose (Sigma, A2095) and immunoblotted according to the methods described above. To detect endogenous polyUb-conjugated NS2, H1299 cells were transfected with pV5-Ub. At 24 h after transfection, cells were infected with A/Puerto Rico/8/34 (H1N1) strain at an MOI of 2 for 0, 6, 9, 12, and 18 h. A lysate was prepared with denaturing IP buffer as described above followed by immunoprecipitation overnight at 4°C with anti-NS2 antibodies (GTX125953, GeneTex) and Protein G agarose beads (Millipore). Immunoprecipitated proteins were detected by immunoblotting according to the methods described above.

### GST and His pulldown assay

*E*. *coli* BL21(DE3)(pGST-OTUB1) and *E*. *coli* BL21(DE3)(pET-NS2) were cultured at 37°C to the mid-log phase. The expression of GST, GST-OTUB1, His-NS2, His, and His-OTUB1 was then induced by treating the cells with 1 mM isopropyl β-D-1-thioglactopyranoside (IPTG) at 37°C for 3 h. After induction, bacterial cells were homogenized with a mini-bead beater (BioSpec Products) in NETN buffer (20 mM Tris-HCl pH 8.0, 1 mM EDTA, 20 mM NaCl, and 0.5% NP40) or lysed in a lysis buffer containing 30 mM NaCl, 50 mM Na_2_HPO4, 0.1% Triton X-100, and 10 mM imidazole. Subsequently, Glutathione-Sepharose 4B beads (Amersham Biosciences) were added to lysates containing GST or GST-OTUB1 and His-NS2. Meanwhile, Ni^2+^-NTA agarose beads (Qiagen) were added to lysates to allow the binding of His or His-NS2 and GST-OTUB1. After 1 h of mixing at 4°C, Glutathione-Sepharose 4B beads were washed extensively with NETN buffer containing 0.5% NP-40; Ni^2+^-NTA agarose beads were washed three times with wash buffer (30 mM NaCl, 50 mM Na_2_HPO_4_, 10 mM imidazole, and 1% Triton X-100). Proteins binding to the beads were then eluted with 2× electrophoresis sample buffer and detected by immunoblotting.

### *In vitro* deubiquitination assay

HEK293T cells were cotransfected with pHA-NS2 and pcDNA-NV5 or pV5-Ub for 24 h, followed by treatment with 5 μM MG132 for 14 h. HA-NS2 and Ub-conjugated HA-NS2 in the lysate were prepared with denaturing IP buffer as described above, and immunoprecipitated with anti-HA agarose beads (Sigma, A2095). Purified His or His-OTUB1 from *E*. *coli* was added and incubated with HA-NS2 or V5-Ub-conjugated HA-NS2 bound to agarose beads in 40 μl deubiquitination buffer (50 mM Tris-HCl at pH 8.0, 5 mM MgCl_2_, 1 mM DTT, and 1 mM ATP) at 37°C for 4 h. The reaction was terminated by adding 5× electrophoresis sample buffer (300 mM Tris-HCl pH 6.8, 5% β-mercaptoethanol, 10% SDS, 50% glycerol, 0.5% NaN_3_, and 0.05% bromophenol blue). Proteins were then analyzed by immunoblotting.

### Determining the half-life of NS2

At 36 h post-transfection, cells were treated with 20 μg/ml cycloheximide and then lysed according to a method described earlier [39–41]. Proteins in the lysate were analyzed by immunoblotting as described previously [38]. The intensity of the protein bands from immunoblotting results was determined by ImageJ (US National Institutes of Health and the Laboratory for Optical and Computational Instrumentation).

### Detecting NA vRNP in the cytoplasm

Cytosolic fraction was prepared using cytosolic extraction buffer (CEB) (Invent Biotech, INC.) according to the manufacturer’s instructions. Proteins in the fraction were analyzed by immunoblotting; RNA was extracted with Trizol reagent (Thermo Fisher). The amount of NA vRNA in the cytoplasm was determined by RT-qPCR.

### Immunofluorescence

A549 or Vero-E6 cells were grown on coverslips and transfected with siRNA using TransIT-X2 Dynamic Delivery System (Mirus Bio). At 24 h after transfection, cells were infected with A/Puerto Rico/8/34 (H1N1) strain at an MOI of 2. For immunostaining, cells were fixed with 4% paraformaldehyde for 20 min, and permeabilized with 0.1% Triton X-100 in PBS for 5 min. After permeabilization, cells were blocked with 1% bovine serum albumin (BSA) in PBS for 1 h at room temperature, and then stained with anti-NP or anti-NS2 antibody (GeneTex). After washing with PBS, cells were blocked with 1% BSA in PBS for 30 min and then incubated with goat anti-rabbit IgG (H+L) conjugated with Alexa Fluor 488 (Invitrogen) for 1 h at room temperature. After staining with 4’,6-diamidino-2-phenylindole (DAPI) or Hoechst 33342, cells were mounted with CitiFluor AF1 (Agar Scientific) and observed under a Nikon TiE eclipse inverted microscope (Nikon).

### Quantifying mRNA, cRNA, and vRNA by RT-qPCR

RNA was isolated from cells using TRIzol Reagent according to the manufacturer’s instructions (Thermo Fisher). After removing genomic or plasmid DNA with a gDNA wiper kit (Vazyme Biotech), RNA was reverse transcribed using a HiScript III 1st stranded cDNA synthesis kit (Vazyme Biotech), which attaches a tagged strand-specific primer capable of annealing to mRNA, cRNA, or vRNA of NA of A/Puerto Rico/8/34 (H1N1) or PB1 of A/Hong Kong/8/68 (H3N2), respectively (S1 Table). RT-qPCR was performed using KAPA SYBR FAST qPCR Master Mix (Merck) with tag primers that respectively bind to the above-mentioned primers specific to mRNA, cRNA, or vRNA of NA of A/Puerto Rico/8/34 (H1N1) or PB1 of A/Hong Kong/8/68 (H3N2) (S1 Table). Primers were designed according to a method described by Kawakami *et al*. [42].

### Plaque assay

MDCK cells (8 × 10^5^) in 6-well dishes were cultured for 24 h and then washed with PBS. IAV A/Puerto Rico/8/34 (H1N1) and A/Hong Kong/8/68 (H3N2) were serially diluted and titered according to a method described by Gaush *et al*. [43]. Plaques were enumerated after staining with crystal violet-formaldehyde solution (0.5% crystal violet in 20% ethanol and 4% formaldehyde).

## Results

### NS2 is ubiquitinated

**Fig 1:**
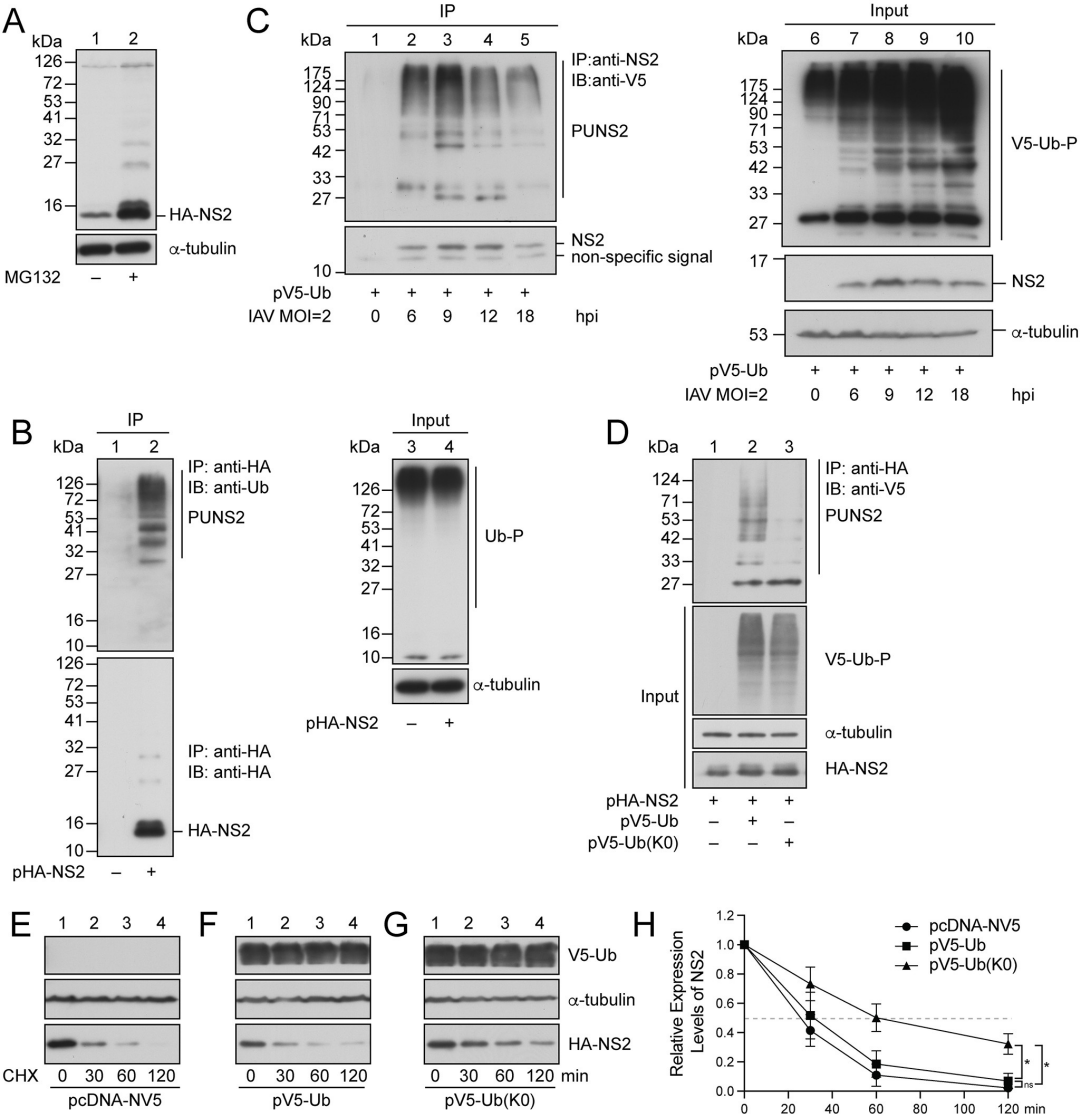
Ubiquitination and instability of NS2. **(A)** HEK293T cells were transfected with pHA-NS2. At 24 h post-transfection, cells were treated with DMSO (lane 1) or MG132 (lane 2) for 14 h. Proteins in the lysates were then detected by immunoblotting with anti-HA and anti-α-tubulin antibodies. **(B)** HEK293T cells were transfected with pcDNA-HA (lane 1) or pHA-NS2 (lane 2), and then treated with MG132. Proteins in the lysate were immunoprecipitated (IP) with anti-HA antibody and immunoblotted (IB) with anti-Ub (upper panel) and anti-HA (lower panel) antibodies (lanes 1–2). **(C)** H1299 cells were transfected with pV5-Ub. At 24 h post-transfection, cells were infected with A/Puerto Rico/8/1934 (H1N1) at an MOI of 2 for 0 h (lanes 1, 6), 6 h (lanes 2, 7), 9 h (lanes 3, 8), 12 h (lanes 4, 9), and 18 h (lanes 5,10). Proteins in the lysates were immunoprecipitated with anti-NS2 antibody and immunoblotted with anti-V5 and anti-NS2 to determine ubiquitinated NS2 and non-ubiquitinated NS2, respectively (lanes 1–5). **(D)** HEK293T cells were cotransfected with pHA-NS2 and pcDNA-NV5 (lane 1), pHA-NS2 and pV5-Ub (lane 2), or pHA-NS2 and pV5-Ub(K0) (lane 3). At 24 h after transfection, cells were treated with MG132 for 14 h. Proteins in the lysates were immunoprecipitated (IP) with anti-HA antibody and immunoblotted (IB) with anti-V5 antibody to show ubiquitinated HA-NS2. The input (B, lanes 3–4; C, lanes 6–10; D, lanes 1–3, lower panel) shows the amount of total ubiquitinated proteins (Ub-P) (B), V5-Ub conjugated proteins (V5-Ub-P) (C, D), NS2 (C), HA-NS2 (D), and α-tubulin (B-D) in the lysates as detected by immunoblotting with anti-Ub, anti-V5, anti-NS2, anti-HA, and anti-α-tubulin antibodies, respectively. PUNS2: polyubiquitinated HA-NS2 (B and D) or NS2 (C). “–” represents an empty vector, pcDNA-HA (A, B) and pcDNA-NV5 (D). **(E-H)** HEK293T cells were cotransfected with **(E)** pHA-NS2 and pcDNA-NV5, **(F)** pHA-NS2 and pV5-Ub, or **(G)** pHA-NS2 and pV5-Ub(K0). At 36 h post-transfection, cells were treated with cycloheximide (CHX). Proteins in the lysate were detected by immunoblotting with anti-HA, anti-V5, and anti-α-tubulin antibodies at 0, 30, 60, and 120 min after the treatment. **(H)** Intensity of the HA-NS2 bands in (E-G) was measured with ImageJ and normalized to the intensity of the respective control α-tubulin band. The amount of NS2 at Hour 0 was set to 1. Error bars represent standard error, which was calculated from the results of three independent sets of experiments. *p < 0.05.

NS2 is known to be present in the cell in low abundance after IAV infection [9]. It is generally believed that inefficient NS mRNA splicing is responsible for the low abundance of NS2 [16]. To elucidate the processes affecting NS2 protein abundance, we transfected HEK293T cells with pHA-NS2. At 24 h after transfection, cells were treated with dimethyl sulfoxide (DMSO) or a proteasome inhibitor, MG132, for 14 h. Immunoblotting with anti-HA antibody revealed that the addition of MG132 greatly increased levels of HA-NS2 (Fig 1A), suggesting that NS2 is degraded by proteasomes. Notably, a band slightly larger than HA-NS2 at the position of 16 kDa is likely phosphorylated HA-NS2 [44]. Furthermore, several faint protein bands larger than HA-NS2 were detected by immunoblotting after MG132 treatment (Fig 1A, lane 2), implying that HA-NS2 undergoes post-translational modification. The observed increases in HA-NS2 levels were not due to variations in sample loading, as control α-tubulin levels remained at consistent levels (Fig 1A). We used a denaturing immunoprecipitation study to determine if NS2 is conjugated with ubiquitin. A lysate was prepared from HEK293T cells that were transfected with pHA-NS2 and then treated with MG132. Proteins in the lysate were immunoprecipitated and immunoblotted with anti-HA. The results revealed an HA-NS2 band migrating to the 14-kDa position (Fig 1B, lane 2, lower panel), although HA-NS2 has an estimated size of 16.9 kDa. When anti-Ub antibody was used in immunoblotting after immunoprecipitation, a series of polyubiquitinated HA-NS2 bands was observed (Fig 1B, lane 2, upper panel). These bands were undetected in the lysate from cells that were not transfected with pHA-NS2 (Fig 1B, lane 1), indicating that NS2 undergoes ubiquitination. Immunoblotting of the proteins in the lysate with anti-Ub antibody revealed that the total amount of ubiquitinated proteins was about equal between samples (Fig 1B, lanes 3–4, upper panel). To determine if NS2 is ubiquitinated after IAV infection, H1299, a non-small cell lung carcinoma cell line, was transfected with pV5-Ub. At 24 h after transfection, cells were infected with A/Puerto Rico/8/34 (H1N1) strain at an MOI of 2. At 0, 6, 9, 12, and 18 h after infection, a lysate was prepared and proteins in the lysate were immunoprecipitated with anti-NS2 antibody and subsequently detected by immunoblotting with anti-V5 antibody. A series of polyubiquitinated NS2 bands began to accumulate after 6 h of infection and reached a maximum level at 9 h after infection, followed by a gradual decline (Fig 1C, lanes 2–5), showing that NS2 is ubiquitinated after IAV infection.

To test if polyubiquitination destabilizes NS2, we first cotransfected HEK293T cells with pHA-NS2 and pV5-Ub or pV5-Ub(K0), which encodes a Ub with all the lysine residues substituted by arginine. At 24 h after cotransfection, cells were treated with MG132 for 14 h. A lysate was then prepared and proteins in the lysate were immunoprecipitated with anti-HA antibody and detected by immunoblotting with anti-V5 antibody. The results showed that the polyUb chains accumulated on HA-NS2 in the presence of V5-Ub. We also observed faint V5-Ub(K0)-NS2 bands with sizes larger than monoubiquitinated HA-NS2 (Fig 1D, lane 3), likely the result of monoubiquitination of NS2 at multiple K residues or incorporated into the polyUb chain formed by the endogenous ubiquitin. We then cotransfected HEK293T cells with pHA-NS2 and pcDNA-NV5 (Fig 1E), pHA-NS2 and pV5-Ub (Fig 1F), or pHA-NS2 and pV5-Ub(K0) (Fig 1G) to determine the influence of polyubiquitination on NS2’s stability. At 36 h after transfection, cells were treated with 20 μg/ml cycloheximide for 0, 30, 60, and 120 min, and proteins in the lysate were detected by immunoblotting with anti-HA antibody. In cells cotransfected with pHA-NS2 and pcDNA-NV5, HA-NS2 had a half-life of 26.3 min (Fig 1E and 1H), while in cells cotransfected with pHA-NS2 and pV5-Ub, a half-life of 30 min was observed (Fig 1F and 1H). There was no statistically significant difference between the two half-life measurements. On the other hand, the half-life of HA-NS2 increased to 60 min after cells were cotransfected with pHA-NS2 and pV5-Ub(K0) (Fig 1G and 1H) (*p < 0.05), showing that conjugation of polyUb chains on NS2 destabilizes HA-NS2.

### Determining the sites and types of ubiquitination for NS2

**Fig 2:**
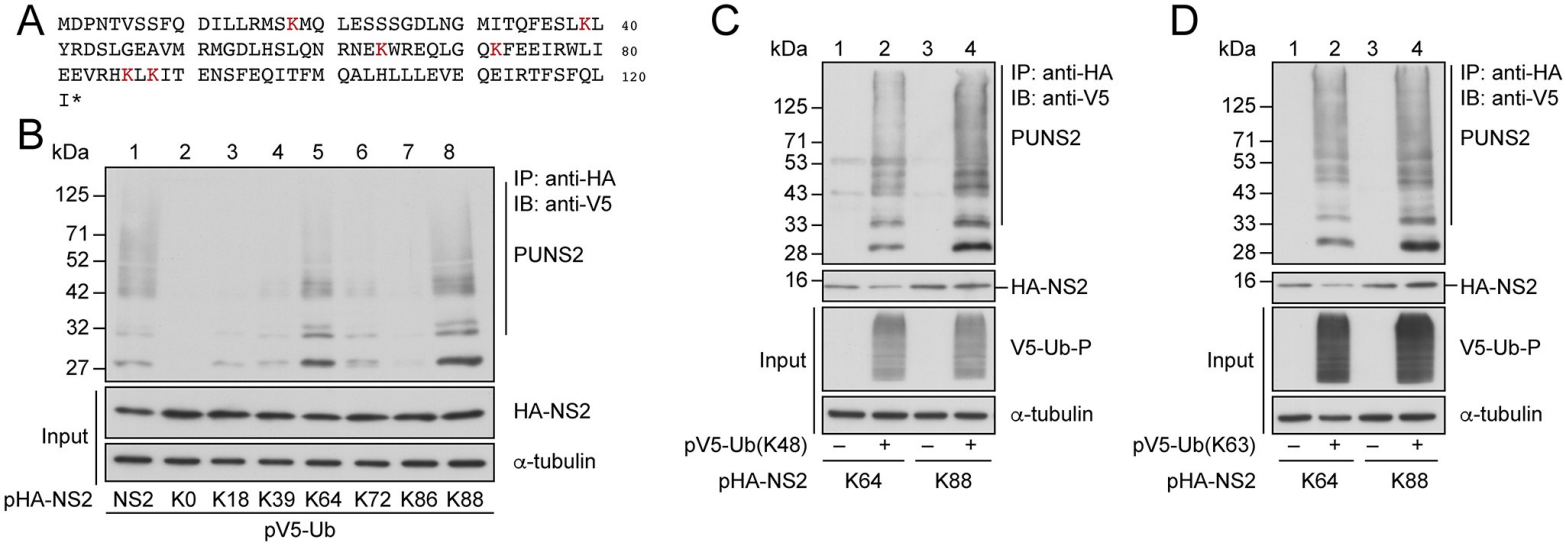
Ubiquitination of HA-NS2. **(A)** The sequence of NS2 from A/Puerto Rico/8/34 (H1N1) strain with its 6 lysine residues highlighted. **(B)** HEK293T cells were cotransfected with pV5-Ub and pHA-NS2 (lane 1), pHA-NS2(K0) (lane 2), pHA-NS2(K18) (lane 3), pHA-NS2(K39) (lane 4), pHA-NS2(K64) (lane 5), pHA-NS2(K72) (lane 6), pHA-NS2(K86) (lane 7), or pHA-NS2(K88) (lane 8). **(C and D)** Cells were cotransfected with pHA-NS2(K64) or pHA-NS2(K88), and either pV5-Ub(K48), pV5-Ub(K63), or pcDNA-NV5. At 24 h after transfection, cells were treated with MG132 for an additional 14 h. Proteins in the lysates were immunoprecipitated (IP) with anti-HA antibody and detected by immunoblotting (IB) with anti-V5 antibody. Immunoblotting with anti-HA, anti-V5, and anti-α-tubulin antibodies was also conducted to indicate the amount of HA-NS2, total ubiquitinated proteins (V5-Ub-P), and α-tubulin in the lysates (Input). PUNS2: polyubiquitinated HA-NS2. “–” represents an empty vector, pcDNA-NV5 (C and D).

NS2 from A/Puerto Rico/8/1934 (H1N1) strain is 121 amino acids in size and contains 6 lysine (K) residues at amino acid positions 18, 39, 64, 72, 86, and 88 (Fig 2A); except for K86, these K residues are highly conserved among human IAV strains (S2 Table). We substituted the 6 lysine residues with arginine to create an HA-NS2(K0) mutant, and then proceeded to generate 6 mutants, HA-NS2(K18), HA-NS2(K39), HA-NS2(K64), HA-NS2(K72), HA-NS2(K86), and HA-NS2(K88), each of which had all lysine residues substituted with arginine, except for the residue indicated. HEK293T cells were cotransfected with plasmids expressing V5-Ub and one of the above HA-NS2 mutants. Proteins in the lysates were immunoprecipitated with anti-HA antibody, followed by immunoblotting with anti-V5 antibody. As expected, HA-NS2(K0) was not ubiquitinated due to the lack of lysine residues (Fig 2B, lane 2). However, HA-NS2, HA-NS2(K64), and HA-NS2(K88) were observed to be conjugated with polyUb chains (Fig 2B, lanes 1, 5, 8). The remaining four HA-NS2 mutants showed evidence of ubiquitination, but at levels substantially lower than HA-NS2(K64) and HA-NS2(K88) (Fig 2B, lanes 3, 4, 6, 7), indicating that the K64 and K88 residues are the predominant sites of ubiquitination for HA-NS2.

Because proteins are commonly conjugated by K48-linked and K63-linked polyUb chains [45,46], we used Ub mutants with all the lysine residues mutated, except for K48 and K63 (respectively labeled as Ub(K48) and Ub(K63) [47–49]), to determine if HA-NS2 is modified by K48-linked and K63-linked polyUb chains. We cotransfected HEK293T cells with pV5-Ub(K48) and pHA-NS2(K64), or pV5-Ub(K48) and pHA-NS2(K88). The result showed that both HA-NS2(K64) and HA-NS2(K88) could be conjugated by K48-linked polyUb chains (Fig 2C, lanes 2, 4). A similar experiment using pV5-Ub(K63) revealed that HA-NS2(K64) and HA-NS2(K88) were conjugated by K63-linked polyUb chains as well (Fig 2D, lanes 2, 4). To ascertain if ubiquitination at K64 and K88 affected the localization of NS2 and nuclear export of vRNP, two IAV mutant strains carrying the NS2(K64) or NS2(K88) were generated with a reverse genetic system. We used anti-NP and anti-NS2 antibodies to examine the distribution of vRNP and NS2, respectively, following infection of A549 cells with IAV carrying wild-type NS2, IAV[NS2(K64)], or IAV[NS2(K88)] at an MOI of 2. At 9 h after infection, NP and NS2 were distributed similarly in the cytosol and the nucleus in the cells infected by IAV, IAV[NS2(K64)], or IAV[NS2(K88)] (S1 Fig). Therefore, ubiquitination at the K64 and K88 residues of NS2 neither alters its distribution nor impacts its function for vRNP nuclear export.

### NS2 interacts with OTUB1

**Fig 3:**
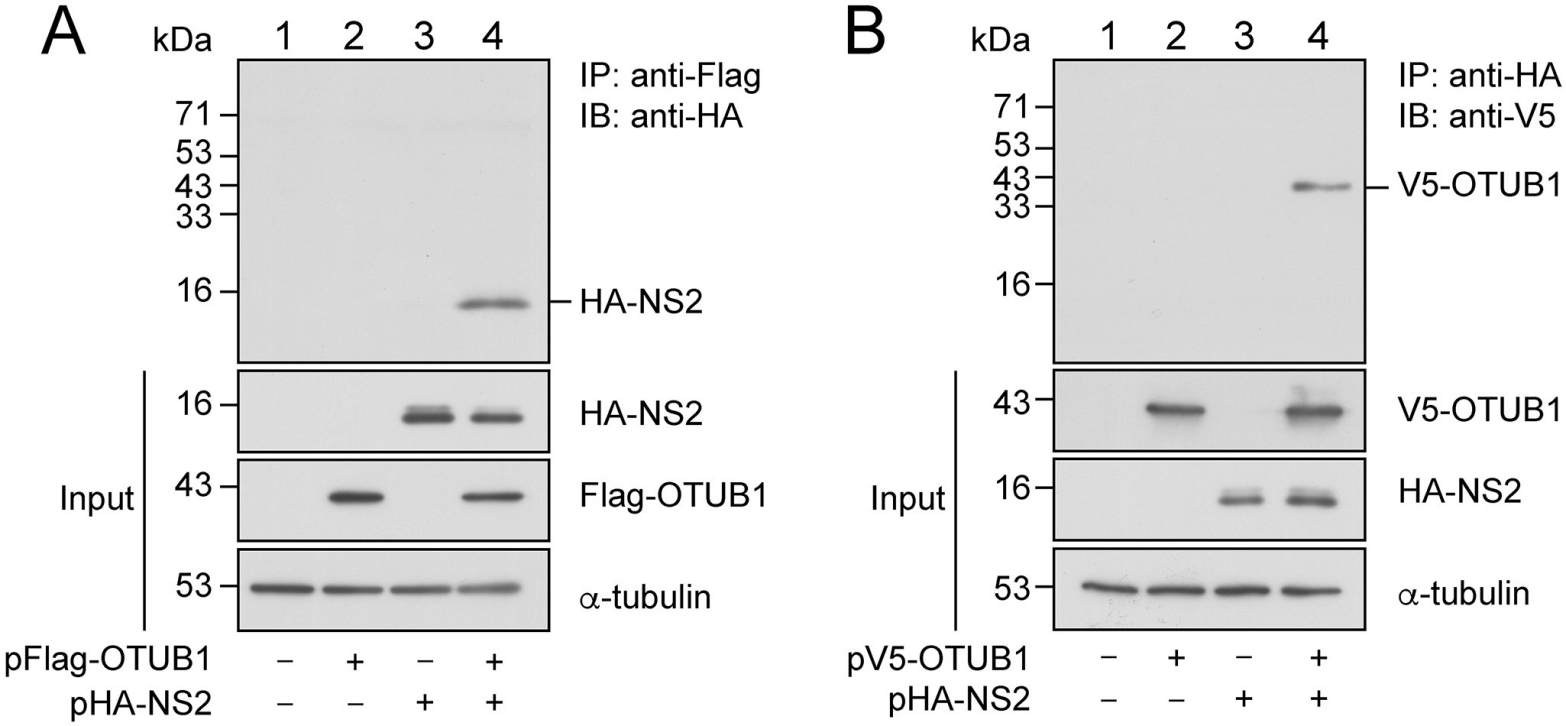
Coimmunoprecipitation of NS2 and OTUB1. **(A)** HEK293T cells were cotransfected with p3XFlag-myc-CMV26 and pcDNA-HA (lane 1); pFlag-OTUB1 and pcDNA-HA (lane 2); p3XFlag-myc-CMV26 and pHA-NS2 (lane 3); pFlag-OTUB1 and pHA-NS2 (lane 4). At 36 h after transfection, proteins in the lysate were immunoprecipitated (IP) with anti-Flag antibody and immunoblotted (IB) with anti-HA antibody. **(B)** HEK293T cells were cotransfected with pcDNA-NV5 and pcDNA-HA (lane 1); pV5-OTUB1 and pcDNA-HA (lane 2); pcDNA-NV5 and pHA-NS2 (lane 3); pV5-OTUB1 and pHA-NS2 (lane 4). At 36 h after transfection, proteins in the lysate were immunoprecipitated with anti-HA antibody and immunoblotted with anti-V5 antibody. Proteins in the lysate (Input) were immunoblotted with anti-HA, anti-Flag, and anti-α-tubulin antibodies (A) or anti-HA, anti-V5, and anti-α-tubulin antibodies (B) without immunoprecipitation. “–” represents an empty vector, p3XFlag-myc-CMV26 or pcDNA-HA (A); pcDNA-NV5 or pcDNA-HA (B).

It is known that NS2 is initially expressed at a low level but accumulates at the late stage of IAV infection, which is important to IAV replication [9]. We therefore searched for mechanisms that may increase the stability of NS2 to foster this accumulation. We conducted a liquid chromatography-mass spectrometry (LC-MS) study to identify proteins that interact with and stabilize NS2. Accordingly, we transfected HEK293T cells with pHA-NS2, and at 30 h after transfection, cells were washed and lysed. Proteins in the lysate were subsequently immunoprecipitated with anti-HA antibody. Immunoprecipitated proteins were then separated by SDS-PAGE and analyzed by LC-MS. Proteins from cells that were transfected with an empty vector were similarly examined. One of the coimmunoprecipitated proteins identified was OTUB1, a deubiquitinase [26,27]. To verify the interaction between NS2 and OTUB1, we cotransfected HEK293T cells with plasmids that expressed HA-NS2 and Flag-OTUB1. At 36 h after transfection, proteins in the cell lysate were immunoprecipitated with anti-Flag M2 agarose beads. Immunoblotting with anti-HA antibody revealed that HA-NS2 was coimmunoprecipitated with Flag-OTUB1 (Fig 3A, lane 4). HA-NS2 was not coimmunoprecipitated by anti-Flag antibody if the cells were not transfected with pFlag-OTUB1 (Fig 3A, lanes, 1, 3) or pHA-NS2 (Fig 3A, lanes 1, 2). In a similar experiment, HEK293T cells were cotransfected with plasmids expressing HA-NS2 and V5-OTUB1. At 36 h after transfection, proteins in the lysate were immunoprecipitated with anti-HA antibody and detected with anti-V5 antibody. The result showed that V5-OTUB1 was coimmunoprecipitated with HA-NS2 (Fig 3B, lane 4), demonstrating that OTUB1 interacts with NS2 in HEK293T cells.

**Fig 4:**
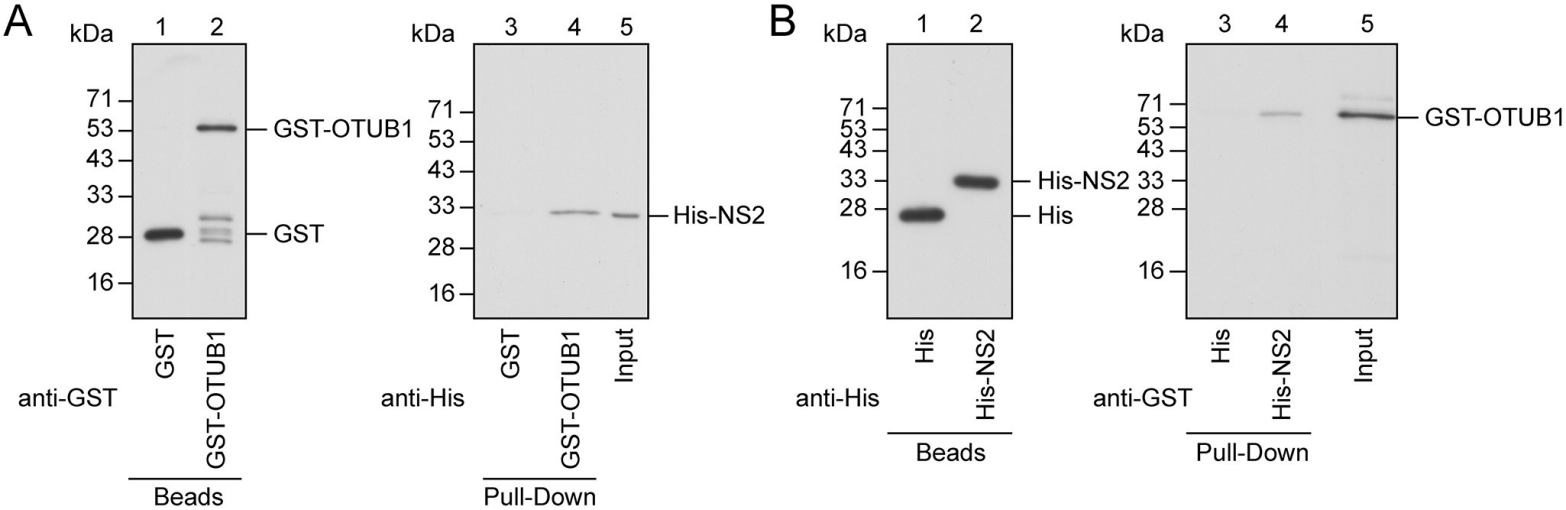
NS2 interacts with OTUB1 *in vitro*. **(A)** GST-glutathione-Sepharose beads (lane 1) or GST-OTUB1-glutathione-Sepharose beads (lane 2) were mixed with bacterially expressed His-NS2. **(B)** His-Ni^2+^-NTA-agarose (lane 1) and His-NS2-Ni^2+^-NTA-agarose beads (lane 2) were mixed with bacterially expressed GST-OTUB1. Proteins pulled down by the beads were detected by immunoblot analysis using the antibodies indicated. Input lanes show His-NS2 (A, lane 5) and GST-OTUB1 (B, lane 5) in the bacterial lysate.

It is known that OTUB1 cleaves polyUb chains from a protein substrate either through its catalytic activity via direct interaction, or non-canonical interference by preventing the interaction of E2-protein substrates [26–29]. We used a GST-pulldown assay to evaluate the interaction between OTUB1 and NS2 *in vitro*. Glutathione-Sepharose beads were added to the lysates from *E*. *coli* BL21(DE3)(pGEX-4T1) and *E*. *coli* BL21(DE3)(pGST-OTUB1) to allow the binding of GST and GST-OTUB1 to the beads, respectively (Fig 4A, lanes 1, 2). In addition, a lysate from *E*. *coli* BL21(DE3)(pET-NS2), which contained NS2 fused to a peptide containing a His-tag, an S-tag, and a Trx-tag thioredoxin (His-NS2; 32 kDa), was subsequently mixed with GST-OTUB1-glutathione-Sepharose or GST-glutathione-Sepharose beads. Immunoblotting with anti-His antibody revealed that GST-OTUB1-Sepharose beads, but not GST-Sepharose beads, pulled down His-NS2 (Fig 4A, lanes 3, 4). A pulldown assay was also conducted with His-NS2-Ni^2+^-NTA agarose beads. We found that the His-NS2-Ni^2+^-NTA agarose beads pulled down GST-OTUB1 (Fig 4B, lane 4); however, GST-OTUB1 was not pulled down by His-Ni^2+^-NTA agarose beads, (Fig 4B, lane 3). Thus, OTUB1 binds NS2 directly *in vitro*.

### OTUB1 deubiquitinates NS2 in cells

**Fig 5:**
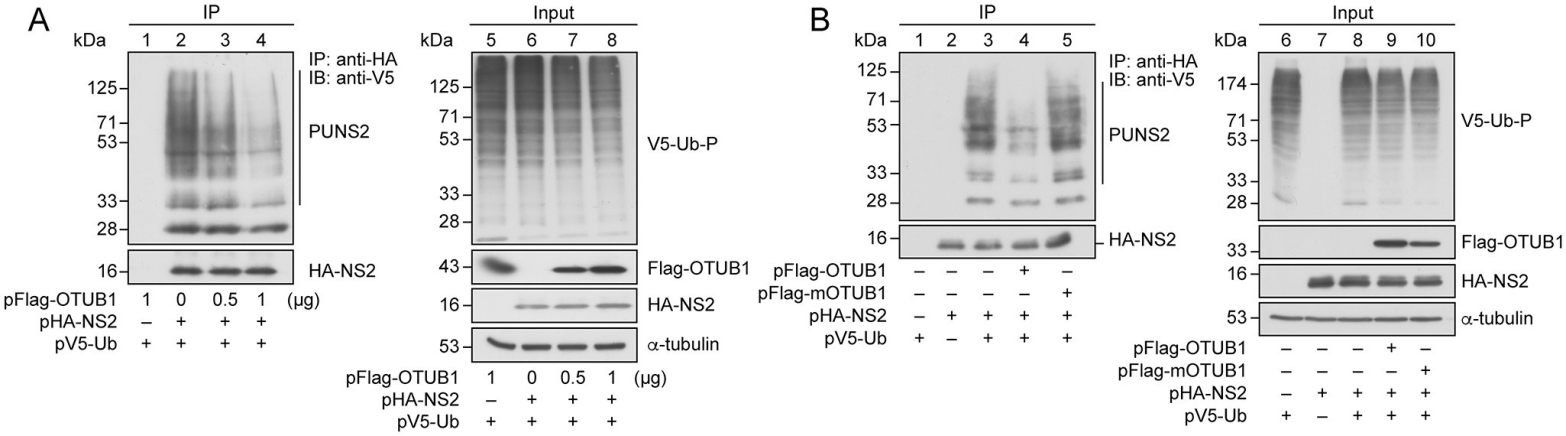
Reduction of NS2 ubiquitination by OTUB1 in HEK293T cells. HEK293T cells were cotransfected with **(A)** pcDNA-HA, pV5-Ub, and pFlag-OTUB1 (lanes 1, 5), or pHA-NS2 and pV5-Ub with varying amounts of pFlag-OTUB1 as indicated (lanes 2–4 and 6–8). At 24 h after transfection, cells were treated with MG132 for 14 h. Polyubiquitinated NS2 in the cell lysate was immunoprecipitated (IP) with anti-HA antibody and detected by immunoblotting (IB) with ant-V5 antibody. “–” represents an empty vector pcDNA-HA. **(B)** Cells were transfected with pV5-Ub (lanes 1, 6), pHA-NS2 (lanes 2, 7), or cotransfected with pHA-NS2 and pV5-Ub (lanes 3, 8), pFlag-OTUB1, pHA-NS2, and pV5-Ub (lanes 4, 9), or pFlag-mOTUB1, pHA-NS2, and pV5-Ub (lanes 5, 10). “–” represents an empty vector, p3XFlag-myc-CMV26, pcDNA-HA, or pcDNA-NV5. At 24 h after transfection, cells were treated with MG132 for 14 h. Proteins in the lysates were immunoprecipitated (IP) with anti-HA antibody and detected by immunoblotting (IB) with anti-HA or anti-V5 antibody (A, lanes 1–4; B, lanes 1–5). The input (A, lanes 5–8; B, lanes 6–10) shows the amount of V5-Ub conjugated protein (V5-Ub-P), HA-NS2, Flag-OTUB1, and α-tubulin in the lysates as detected by immunoblotting with anti-V5, anti-HA, anti-Flag, and anti-α-tubulin antibodies, respectively. PUNS2: polyubiquitinated HA-NS2.

Considering that OTUB1 is a deubiquitinase [26,27], we asked if OTUB1 deubiquitinates and stabilizes NS2. HEK293T cells were cotransfected with pHA-NS2, pV5-Ub, and 0.5 μg or 1 μg pFlag-OTUB1. Cells were then treated with MG132; proteins in the lysate were immunoprecipitated and immunoblotted with anti-HA and anti-V5 antibodies, respectively (Fig 5A, lanes 1–4). The level of polyubiquitinated HA-NS2 was reduced after the expression of Flag-OTUB1 in a dose-dependent manner (Fig 5A, lanes 2–4) while the controls, α-tubulin, HA-NS2, and total ubiquitinated protein, in each lane were unaffected (Fig 5A, lanes 5–8), indicating that the reduction of polyubiquitinated HA-NS2 was not due to sample loading variations or lack of HA-NS2 expression. A similar experiment was conducted by transfecting pFlag-mOTUB1, which encodes an OTUB1 mutant with D88A and C91S substitutions. These mutations are known to eliminate the deubiquitinase activity of OTUB1 [26]. We found that although levels of NS2 conjugated with V5-Ub were substantially reduced if HEK293T cells were cotransfected with pFlag-OTUB1 (Fig 5B, lanes 3, 4), no reduction of Ub-HA-NS2 was observed if cells were cotransfected with pFlag-mOTUB1 (Fig 5B, lane 5), indicating that OTUB1 deubiquitinates NS2 through its catalytic activity.

### Deubiquitination of HA-NS2 by His-OTUB1 *in vitro*

**Fig 6:**
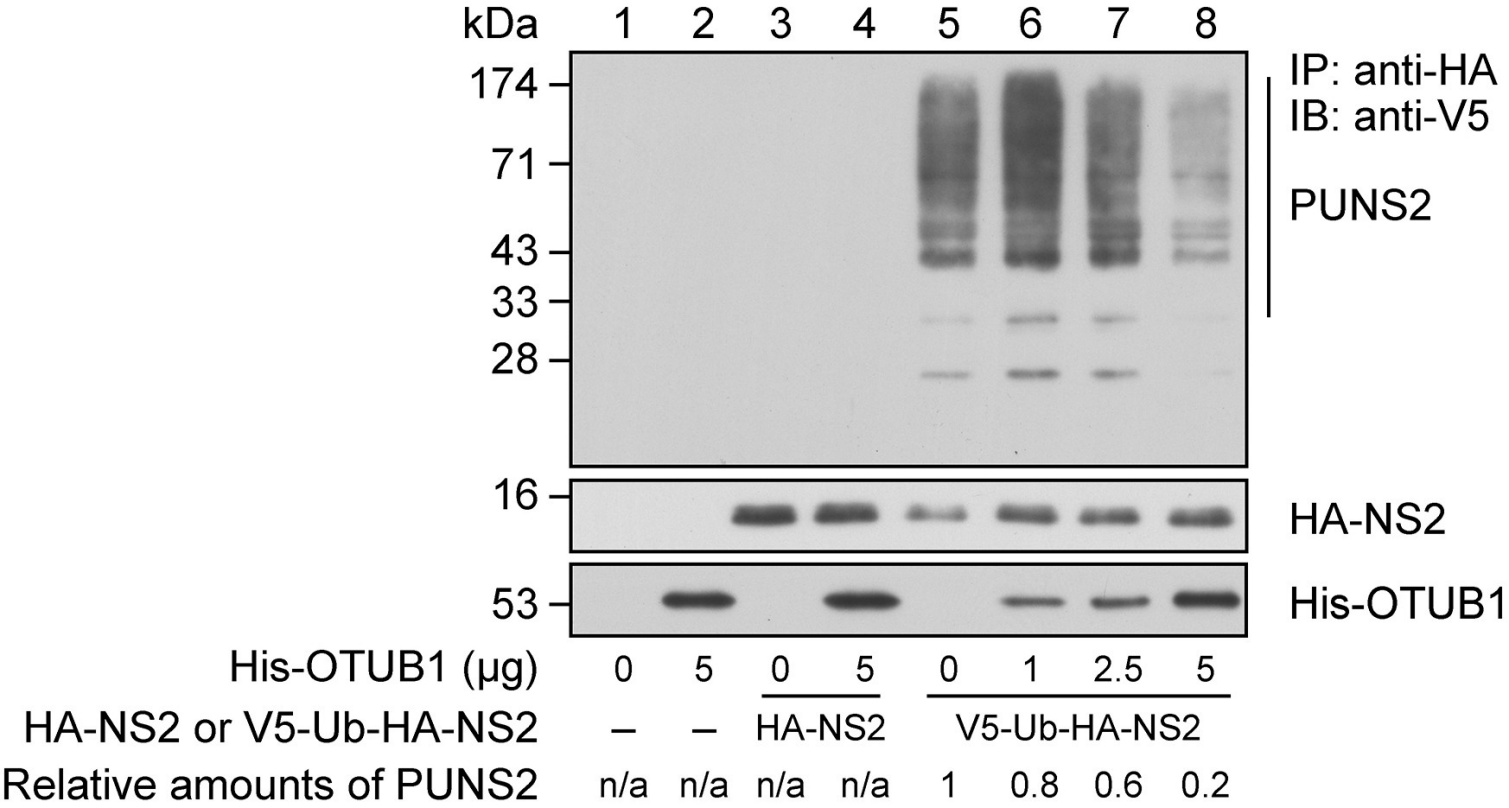
Deubiquitination of ubiquitinated HA-NS2 by His-OTUB1 *in vitro*. HA-NS2 (lanes 3, 4) and HA-NS2 conjugated with V5-Ub (V5-Ub-HA-NS2) (lanes 5–8) were purified by immunoprecipitation using anti-HA antibody from HEK293T cells that had been transfected with pHA-NS2 or cotransfected with pV5-Ub and pHA-NS2, respectively. Proteins immunoprecipitated (IP) from cells cotransfected with pV5-Ub and pcDNA-HA in a similar way were used as a negative control (lanes 1, 2). Immunoprecipitated proteins were then mixed with varying amounts of bacterially-expressed His-OTUB1 as indicated. After deubiquitination, polyubiquitinated HA-NS2 (V5-Ub-HA-NS2; PUNS2) was detected by immunoblotting (IB) with anti-V5 antibody. The amount of HA-NS2 and OTUB1 in the reaction mixture was analyzed by immunoblotting with the antibodies indicated. The amount of polyubiquitinated NS2 was normalized to immunoprecipitated NS2 levels. The intensity ratio between PUNS2 and HA-NS2 bands in the absence of His-OTUB1 (lane 5) was set as 1. “–” represents an empty vector, pcDNA-HA.

To confirm that OTUB1 can deubiquitinate NS2 *in vitro*, we immunoprecipitated HA-NS2 from HEK293T cells that were transfected with pV5-Ub or pHA-NS2, or cotransfected with both plasmids. HA-NS2 and HA-NS2 that was conjugated by V5-Ub (V5-Ub-HA-NS2) were purified by immunoprecipitation with anti-HA antibody and used as substrates for deubiquitination by bacterially-expressed His-OTUB1. As expected, V5-Ub-HA-NS2 was undetected in the reaction mixture if the substrate was prepared from cells transfected with only pV5-Ub (Fig 6, lanes 1, 2) or pHA-NS2 (Fig 6, lanes 3, 4). Polyubiquitinated HA-NS2 by V5-Ub was detected in substrates prepared from cells cotransfected with pHA-NS2 and pV5-Ub (Fig 6, lanes 5–8). We found that 1–5 μg bacterially-expressed His-OTUB1 reduced V5-Ub-HA-NS2 (Fig 6, lanes 6–8). Although the polyubiquitinated HA-NS2 (V5-Ub-HA-NS2) bands in the reaction containing 1 μg His-OTUB1 (Fig 6, lane 6) were more intense than the bands from the reaction without His-OTUB1 (Fig 6, lane 5), we attribute this to sample loading, as the amount of HA-NS2 was less in lane 5 than lane 6 (Fig 6, lanes 5, 6). The results showed that the amount of polyubiquitinated HA-NS2 decreased in a dose-dependent manner as 1–5 μg His-OTUB1 were added to the reaction mixture.

### OTUB1 stabilizes NS2

**Fig 7:**
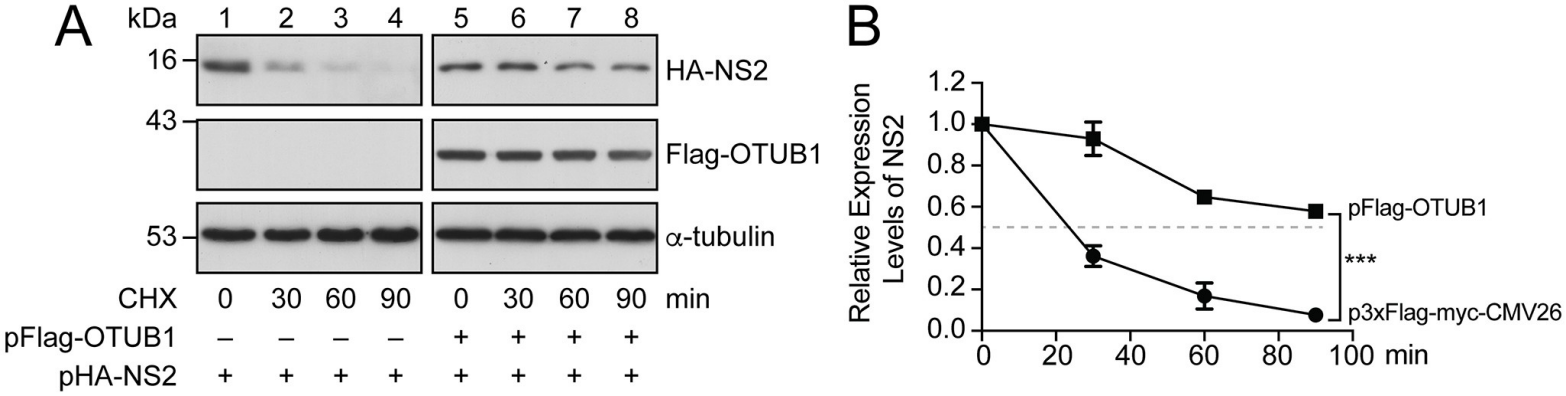
Stabilization of NS2 by OTUB1. **(A)** HEK293T cells were cotransfected with pHA-NS2 (lanes 1–8) and an empty vector, p3XFlag-myc-CMV26 (lanes 1–4) or pFlag-OTUB1 (lanes 5–8). At 36 h post-transfection, cells were treated with cycloheximide (CHX). Proteins in the lysate were detected by immunoblotting with anti-HA, anti-Flag, and anti-α-tubulin antibodies. Levels of α-tubulin in the lysates were used as a loading control. **(B)** The intensity of the protein bands in (A) was measured with ImageJ, and normalized to the intensity of the respective control α-tubulin band. The amount of NS2 at Hour 0 was set to 1. The error bar represents standard error, which was calculated from the results of three independent sets of experiments. ***p < 0.001. “–” represents an empty vector, p3XFlag-myc-CMV26.

We cotransfected HEK293T cells with pHA-NS2 and p3xFlag-myc-CMV26 or pHA-NS2 and pFlag-OTUB1 to determine whether the expression of OTUB1 stabilizes HA-NS2. At 36 h after cotransfection, cells were treated with 20 μg/ml cycloheximide for 0, 30, 60, or 90 min. A lysate was prepared, and proteins in the lysate were analyzed by immunoblotting with anti-HA antibody. In cells cotransfected with pHA-NS2 and p3xFlag-myc-CMV26, the amount of HA-NS2 decreased rapidly (Fig 7A, lanes 1–4), with a half-life of about 23.4 min (Fig 7B). On the other hand, in cells cotransfected with pHA-NS2 and pFlag-OTUB1, HA-NS2 remained relatively stable, with a half-life of more than 90 min (Fig 7A, lanes 5-8; Fig 7B). This indicates that the expression of Flag-OTUB1 stabilizes HA-NS2.

### OTUB1 promotes the replication of IAV

**Fig 8:**
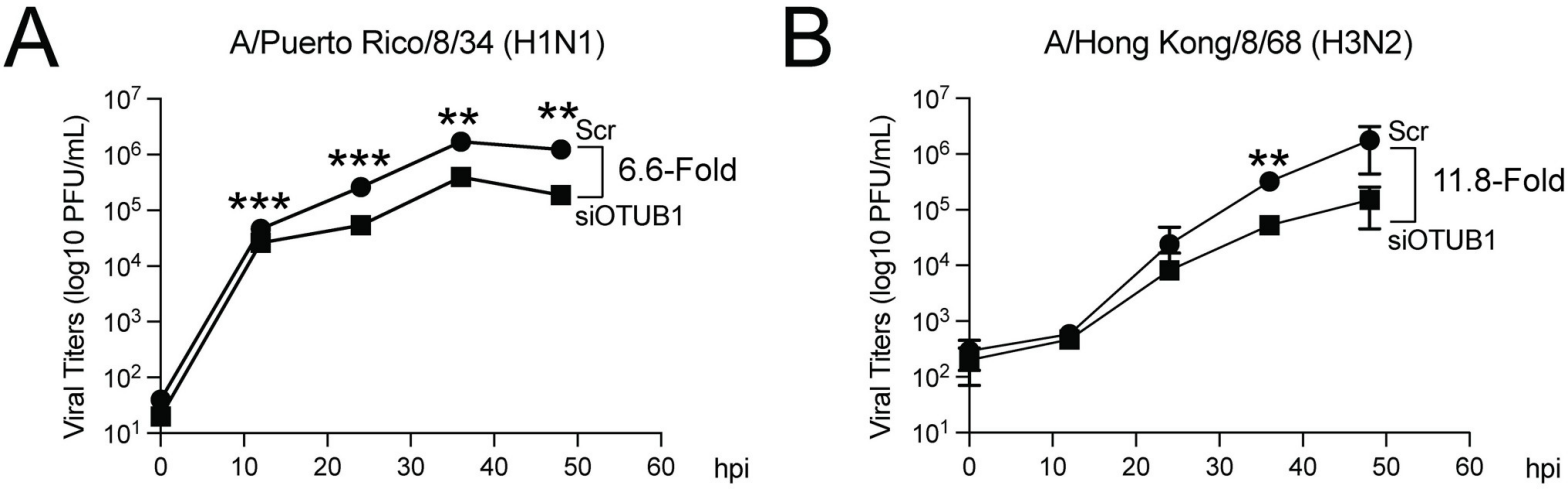
Reduction of OTUB1 decreased IAV progeny production. Virus particles released from Vero-E6 cells transfected with OTUB1 siRNA or scramble siRNA and then infected with the A/Puerto Rico/8/1934 (H1N1) strain **(A)** or A/Hong Kong/8/68 (H3N2) strain **(B)** at an MOI of 0.01. At 0 h, 12 h, 24 h, 36 h, and 48 h after infection, the supernatant was collected and viral load was enumerated by plaque assays. Error bars represent standard error, which was calculated from the results of three independent sets of experiments. **p < 0.01; ***p <0.001.

We also examined how OTUB1 siRNA knockdown affects IAV proliferation. After transfecting Vero-E6 cells with OTUB1 siRNA, cells were infected with IAV at an MOI of 0.01 to allow multiple rounds of infection to occur during a 48-hour period. Plaque assay results showed that OTUB1 siRNA knockdown reduced plaque-forming units (PFU) of the A/Puerto Rico/8/1934 (H1N1) strain by 6.6-fold at 48 h after infection (Fig 8A). Furthermore, a 11.8-fold reduction on PFU was also observed for A/Hong Kong/8/68 (H3N2) strain (Fig 8B). Similar results were observed with experiments conducted in A549 cells (S2 Fig). In order to explain this important insight, we examined how OTUB1 affects the replication of IAV.

### OTUB1 contributes to vRNP export

**Fig 9:**
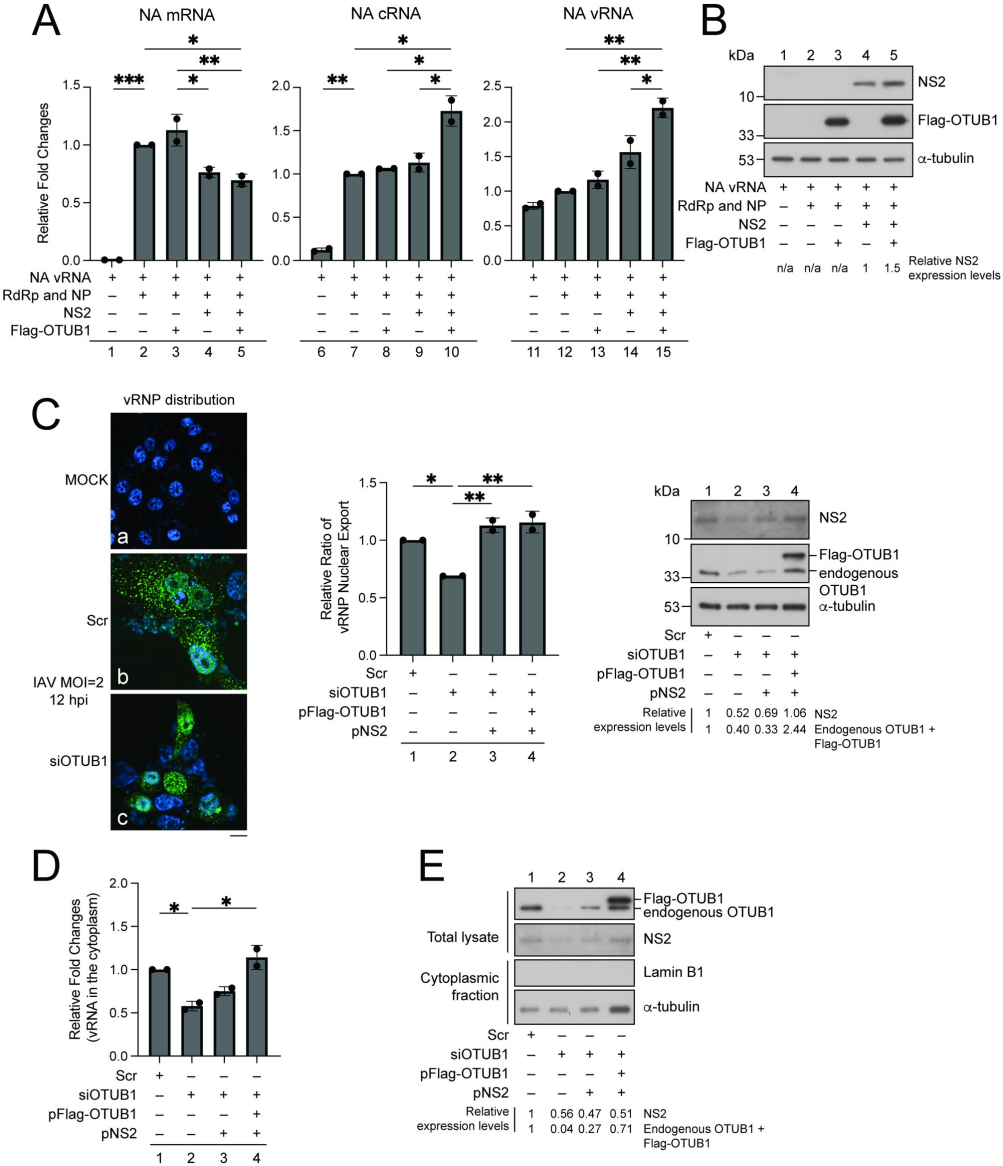
Influence of OTUB1 on IAV transcription, RNA replication, and vRNP nuclear export. **(A-B)** HEK293T cells were transfected with pHH-NA (columns 1, 6, 11), or cotransfected with pHH-NA, pHW-NP, and RdRp plasmids (columns 2, 7, 12); pHH-NA, pHW-NP, RdRp plasmids, and pFlag-OTUB1 (columns 3, 8, 13); pHH-NA, pHW-NP, RdRp plasmids, and pNS2 (columns 4, 9, 14); and pHH-NA, pHW-NP, RdRp plasmids, pFlag-OTUB1, and pNS2 (columns 5, 10, 15). At 36 h after transfection, levels of NA mRNA, cRNA, and vRNA were determined by RT-qPCR **(A)**. The amount of mRNA, cRNA, and vRNA in the cells cotransfected with pHH-NA, RdRp plasmids, pHW-NP was set to 1 (columns 2, 7, 12). “-” represents an empty vector, pcDNA3.1(+). **(B)** HEK293T cells were similarly transfected and cotransfected as described above. Proteins in the lysate were examined by immunoblotting with anti-α-tubulin, anti-NS2, or anti-Flag antibodies. The intensity of the NS2 band in lanes 4 and 5 was measured using ImageJ, and normalized against the intensity of α-tubulin bands. Results are presented as fold change relative to lane 4. “–” represents the indicated proteins were not expressed in HEK293T cells **(C)** Vero-E6 cells were transfected with scramble siRNA (b), OTUB1 siRNA (c) or without transfection (a). At 24 h after transfection, cells were mock-infected (a) or infected with the A/Puerto Rico/8/1934 (H1N1) strain of IAV (b-c) at an MOI of 2. At 12 h after infection, vRNP was stained by indirect immunofluorescence with anti-NP and goat anti-rabbit antibody conjugated with Alexa Fluor-488 (green). The nucleus was stained with Hoechst 33342 (blue). Representative cell images were deconvoluted with NIS-elements v5.2 (Nikon). Scale bar represents 10 μm. Cells with vRNP nuclear export were enumerated after examining about 350 cells in 20 microscopic fields. Cells were transfected with scramble siRNA (column 1), OTUB1 siRNA (column 2) or cotransfected with OTUB1 siRNA and pNS2 (column 3) to show how the reduction of OTUB1 or NS2 influenced nuclear export. Cells were also cotransfected with OTUB1 siRNA, pFlag-OTUB1, and pNS2 to show how the expression of Flag-OTUB1 and NS2 affected nuclear export (column 4). NS2, OTUB1, and α-tubulin in the cells were also examined by immunoblotting with anti-NS2, anti-OTUB1, and anti-α-tubulin antibodies, respectively. The fractions of cells with vRNP nuclear export (bar chart) and the amount of NS2 or OTUB1 in cells (immunoblot) transfected with scramble siRNA was set to 1. **(D-E)** Vero-E6 cells were transfected with scramble siRNA (column 1), OTUB1 siRNA (column 2), or cotransfected with OTUB1 siRNA and pNS2 (column 3) or OTUB1 siRNA, pFlag-OTUB1, and pNS2 (column 4). After transfection and infection for 9 h, the cytoplasm was fractionated and NA vRNA in the cytoplasm was determined by RT-qPCR **(D)**. The amount of NA vRNA in column 1 was set to 1. (**E)** Proteins in the total lysate or cytoplasm were also examined by immunoblotting with anti-lamin B1, anti-α-tubulin, anti-NS2, or anti-OTUB1 antibodies. Error bars represent standard error, which was calculated from the results of two independent sets of experiments. *p < 0.05; **p < 0.01; ***p <0.001. “–” represents an empty vector, p3XFlag-myc-CMV26, or without either scramble siRNA or OTUB1 siRNA.

As NS2 is known to promote IAV replication and vRNP nuclear export [9,12,13], we asked first if these two steps of the IAV life cycle are influenced by OTUB1-mediated deubiquitination of NS2. HEK293T cells were cotransfected with plasmids encoding subunits of RdRp (pHW-PB2, pHW-PB1, and pHW-PA), pHH-NA, pHW-NP, pNS2, pFlag-OTUB1, or pcDNA3.1(+) as indicated in Fig 9A and 9B. At 36 h after transfection, levels of NA mRNA, cRNA, and vRNA were measured by RT-qPCR. NA mRNA was undetected after the cells were transfected with pHH-NA alone, (Fig 9A, column 1) but the amount of NA mRNA increased substantially after the cells were cotransfected with RdRp plasmids and pHW-NP (Fig 9A, column 2). Although transfecting pFlag-OTUB1 did not influence transcription as activated by RdRp (Fig 9A, column 3), transfecting the cells with pNS2 decreased transcription by 25% (Fig 9A, column 4). Transcription activation declined by 30% if Flag-OTUB1 was also expressed (Fig 9A, column 5). We also examined how NS2 and OTUB1 influenced cRNA synthesis. We found that transfecting the cells with RdRp and NP plasmids increased the amount of cRNA in the cells (Fig 9A, columns 6, 7). However, the expression of NS2 or OTUB1 did not further increase the amount of cRNA (Fig 9A, columns 8, 9). Interestingly, the amount of cRNA increased by 1.7-fold after the cells were cotransfected with pNS2 and pFlag-OTUB1 (Fig 9A, column 10). In the case of vRNA synthesis, we detected NA vRNA without transfecting RdRp and NP plasmids (Fig 9A, column 11) due to the transcription of NA vRNA from a PolI promoter in the plasmid. After the cells were also transfected with RdRp and NP plasmids (Fig 9A, column 12) or RdRp plasmids and pFlag-OTUB1 (Fig 9A, column 13), the amount of NA vRNA in the cells increased slightly. NA vRNA levels increased by 1.6-fold after cotransfection of RdRp plasmids, pHW-NP, and pNS2 (Fig 9A, column 14), and further increased by 2.2-fold after pFlag-OTUB1 was additionally transfected (Fig 9A, column 15). These results suggest that stabilization of NS2 by OTUB1 influences IAV transcription and RNA replication. Furthermore, in an immunoblot analysis, we showed that the expression of Flag-OTUB1 increased the abundance of NS2 (Fig 9B). These results suggest that stabilization of NS2 by OTUB1 influences IAV transcription and RNA replication. We additionally examined the influence of NS2 expression on IAV transcription and viral RNA replication after IAV infection. We knocked down OTUB1 expression in Vero-E6 cells with siRNA, followed by infection with A/Puerto Rico/8/1934 (H1N1) strain or A/Hong Kong/8/68 (H3N2) strain at an MOI of 2. At 4, 8, and 12 h post-infection, we found that OTUB1 knockdown did not affect mRNA, cRNA, and vRNA levels (S3 Fig).

Importantly, we did find that OTUB1 knockdown affected vRNP nuclear export. We introduced scramble siRNA or OTUB1 siRNA into Vero-E6 cells, and then infected the cells with the A/Puerto Rico/8/1934 (H1N1) strain at 24 h after transfection. After examining approximately 350 cells, we found that the majority of cells that stained positive for NP expression had vRNP manifesting as speckles in the cytoplasm at 12 h after IAV infection (Fig 9C, Image, b; Bar chart, column 1). However, cells with vRNP speckles in the cytoplasm dropped by about 30% after the introduction of OTUB1 siRNA (Fig 9C, Image, c; Bar chart, column 2). Additionally, transfecting the cells with pNS2 or both pNS2 and pFlag-OTUB1 followed by IAV infection fully restored vRNP export (Fig 9C, Bar chart, columns 3, 4), suggesting that siRNA knockdown of OTUB1 hampers the nuclear export of vRNP. We proceeded to determine the amount of vRNA in vRNP that was exported to the cytoplasm, by fractionating the cytoplasm from Vero-E6 cells that were infected by IAV for 9 h. We found that introducing OTUB1 siRNA decreased the amount of NA vRNA in the cytoplasm by 42% (Fig 9D, column 2), while transfection of pNS2 partially increased vRNA levels in the cytoplasm to 75% (Fig 9D, column 3). In addition, vRNP nuclear export was fully recovered after the expression of NS2 and Flag-OTUB1 (Fig 9D, column 4). Our results also showed that the cytosolic fraction contains α-tubulin and was not contaminated by lamin B1 nuclear protein (Fig 9E). These results confirm that OTUB1 facilitates the nuclear export of vRNP by NS2.

## Discussion

NS2 is thought to play a pivotal role in regulating IAV cRNA synthesis and vRNP nuclear export [12,13,18,19]. It is known that NS2 is present in low abundance at the early stage of IAV infection, but increases to reach a maximum at the late stage of infection [12,13]. The presence of NS2 in low abundance at the initial stage of infection is known to benefit viral transcription [9], which is critical to IAV proliferation. It is also known that the low abundance of NS2 is attributable to poor splicing of NS mRNA, resulting in low yields of the NS2 transcript [16]. We show here that NS2 destabilization by ubiquitination contributes to its low abundance.

NS2 is a 121-amino acid long with highly conserved lysine (K) residues at amino acid positions 18, 39, 64, 72, and 88 (Fig 2A and S2 Table). We found that NS2 is ubiquitinated (Fig 1B, lane 2 and Fig 1C, lanes 2–5) at the K64 and K88 residues (Fig 2B) by K48-linked and K63-linked polyUb chains (Fig 2C and 2D). Proteins linked with K48-linked polyUb chains are often degraded by proteasomes [45,46], and NS2 is too. Treating cells with MG132, a proteasome inhibitor, substantially increases the abundance of NS2 (Fig 1A). The fact that NS2 is also conjugated by K63-linked polyUb chains suggesting that NS2 is degraded by a proteasome-independent pathway [50] as well (Fig 2D). We found importantly, however, that ubiquitination at the 64^th^ and 88^th^ residues of NS2 did not affect vRNP nuclear export (S1 Fig).

In an LC-MS proteomic investigation, we identified a host DUB, OTUB1, which interacts with viral NS2. The interaction was verified in cells and *in vitro* (Figs 3 and 4). We also showed that overexpressing OTUB1 reduces the amount of polyubiquitinated NS2 (Fig 5A and 5B, lane 4). To verify that OTUB1 is a deubiquitinase of NS2, we mutated the D88 and C91 residues, which are crucial for the deubiquitination activity of OTUB1 [26], to create mOTUB1. We found that mOTUB1 was unable to reduce the amount of polyubiquitinated NS2 (Fig 5B, lane 5). Furthermore, bacterially-expressed His-OTUB1 was able to deubiquitinate polyubiquitinated NS2 *in vitro* (Fig 6, lane 8). Transfection of pFlag-OTUB1 was shown to increase the stability of HA-NS2 (Fig 7), thereby confirming that OTUB1 can deubiquitinate and stabilize NS2. This finding implies that OTUB1 is a key factor in contributing to the accumulation of NS2 at the late stage of IAV infection.

Earlier studies have shown that NS2 levels can influence IAV transcription, replication, and vRNP nuclear export. Indeed, in a transfection study, we verified that the expression of OTUB1 after transfection decreases mRNA transcription but increases cRNA and vRNA synthesis (Fig 9A). However, the expression of NS2 from pNS2 does not seem to influence IAV transcription and RNA replication unless OTUB1 is also expressed (Fig 9A, columns 4, 9). It is likely that the amount of NS2 expressed from pNS2, which expresses the full-length NS transcript, may be low due to poor splicing of the NS mRNA [16], and therefore cannot cause significant IAV RNA transcription and replication changes unless it is stabilized by OTUB1 (Fig 9A). In theory, the knockdown of OTUB1 by siRNA after IAV infection should destabilize NS2, which may affect IAV RNA transcription and replication. However, OTUB1 siRNA knockdown did not detectably affect mRNA, cRNA, and vRNA expression after IAV infection (S3 Fig). This seeming paradox may be explained by the fact that overexpression of NS2 is needed to observe any significant enhancement of cRNA and vRNA syntheses [13], but after IAV infection, NS2 is not naturally expressed at a high level [16], and therefore any decline in already low levels of NS2 caused by OTUB1 siRNA knockdown does not appear to substantially affect levels of cRNA and vRNA in the cells (S3 Fig). In addition, NS2 regulation of IAV RNA transcription and replication is complex [12,13]; in the absence of OTUB1, NS2 is less stable, and although this decreases RNA replication, it can conversely increase transcription, which may heighten the expression of RdRp and lead to increased RNA replication.

NS2 is known to function as an adaptor, forming a Crm1-NS2-M1-vRNP complex to facilitate the nuclear export of vRNP [18;36]. This study demonstrates that reduction of OTUB1 expression by siRNA reduces vRNP nuclear export (Figs 9C, 9D and S2), indicating that OTUB1 plays an important role in IAV infection. We also observed that the reduction of OTUB1 by siRNA ultimately influences IAV proliferation; therefore, OTUB1 may serve to promote IAV progeny production (Fig 8). It is known that OTUB1 activates innate immunity, which may restrict IAV proliferation [32]; therefore, we elected to conduct a set of experiments in Vero-E6 cells, as this cell line is deficient in innate interferon production [51]. Our knocking down the expression of OTUB1 provided the important insight that it decreases virion production by the A/Puerto Rico/8/1934 (H1N1) and A/Hong Kong/8/68 (H3N2) strains by 6.6-fold and 11.8-fold at 48 h after infection, respectively (Fig 8). The stabilization of NS2 by OTUB1 therefore critically influences not only vRNP nuclear export but also virion production.

**Fig 10:**
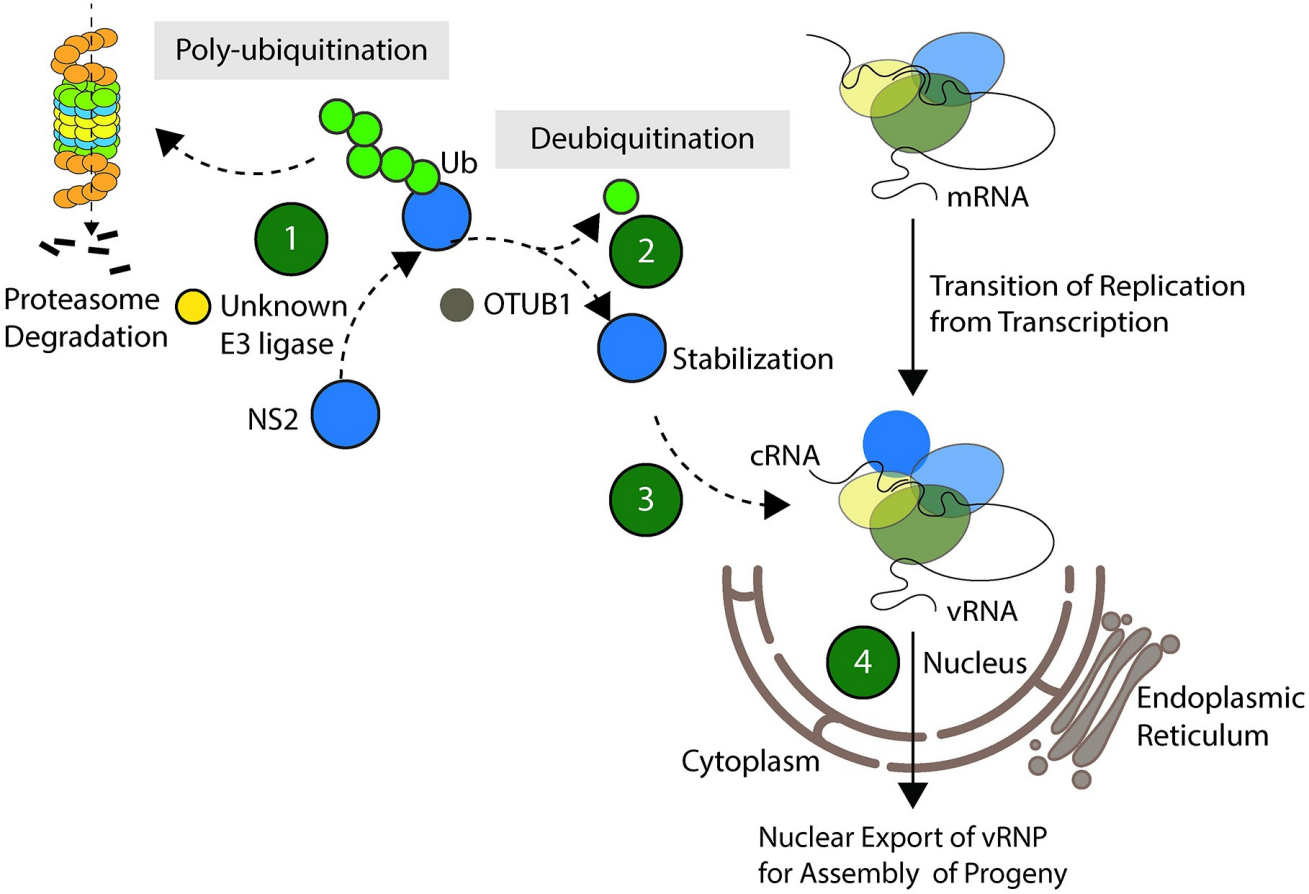
Schematic depicting the viral-host interaction between NS2 and OTUB1 that facilitates IAV propagation as proposed in this study.

OTUB1 has been demonstrated to inhibit IAV infection by activating the antiviral response [32]. After IAV infection, OTUB1 has been observed to translocate from the nucleus to the mitochondrial membrane and subsequently activate RIG-1 through deubiquitination and E2 suppression, thereby triggering RIG-1-dependent immune signaling and antiviral responses [32]. Additionally, the promotion of OTUB1 degradation by IAV NS1 has been reported to be a mechanism by which IAV evades the antiviral response [32]; however, OTUB1 expression levels appear to remain constant throughout the IAV life cycle in our study (S4 Fig). On the other hand, OTUB1 has a seemingly paradoxical function in which it promotes IAV replication, as overexpression of OTUB1 was previously reported to repress virus-triggered interferon induction and other host antiviral responses [33]. The paradox in which OTUB1 appears to promote both an innate anti-IAV immune response as well as IAV proliferation can be explained by the results of our study, which shows that OTUB1 interacts with NS2 to set the stage for viral cRNA synthesis and is critical for vRNP nuclear export (Figs 9 and 10). Knocking down the expression of OTUB1 would affect NS2 accumulation, possibly delaying cRNA synthesis and also preventing efficient vRNP nuclear export, thereby hindering viral propagation (Fig 8). It also seems that although OTUB1 can trigger host antiviral responses [32], overexpression of OTUB1 has been shown to conversely attenuate antiviral responses [33], suggesting a temporal-spatial mode of regulation is likely involved. Our study sheds light on the critical viral-host interaction between NS2 and OTUB1 that facilitates viral propagation (Figs 8 and 10), and further research into the changes in quantity and locality of OTUB1 following IAV infection may be warranted, in order to better understand how IAV utilizes host factors to enhance viral proliferation.

## Supporting information

S1 Fig Distributions of NS2, NS2(K64), and NS2(K88) in IAV infected A549 cells. A549 cells were infected by IAV **(A, a-c; B, a-c)**, IAV[NS2(K64)] **(A, d-f; B, d-f)**, or IAV[NS2(K88)] **(A, g- i; B, g-i)** for 9 h and stained by indirect immunofluorescence using anti-NS2 **(A)** or anti-NP antibody **(B)** followed by goat anti-rabbit antibody conjugated with Alexa Fluor-488 (green). The nucleus was stained with DAPI (blue). Cells were observed under a Nikon TiE Eclipse inverted microscope. Representative cell images were deconvoluted with NIS-elements v5.2 (Nikon). Scale bar: 10 μm.
(EPS)

S2 Fig Reduction of OTUB1 decreased NS2-mediated vRNP nuclear export. **(A-C)** A549 cells were transfected with OTUB1 siRNA or scramble siRNA. After transfection, cells were infected with the A/Puerto Rico/8/1934 (H1N1) strain of IAV at an MOI of 2. At 24 h after transfection, OTUB1 in the cell lysate was analyzed by immunoblotting **(A)**; At 12 h after infection, vRNP in mock-infected cells (a-c) or infected by IAV (d-i) and transfected with scramble siRNA (d, e, f) or OTUB1 siRNA (g, h, i) was stained by indirect immunofluorescence with anti-NP and goat anti-rabbit antibody conjugated with Alexa Fluor-488 (green). The nucleus was stained with DAPI (blue). c, f, i are merged images. Cells were observed under a Nikon TiE Eclipse inverted microscope. The arrow indicates the nuclear export of vRNP. Representative cell images were deconvoluted with NIS-elements v5.2 (Nikon). Scale bars represent 10 μm **(B). (C)** Approximately 150 cells in 12 microscopic fields were examined for the presence of vRNP in the cytoplasm at 6, 10, 12, and 15 h after infection. **(D)** Virus particles released from A549 cells transfected with OTUB1 siRNA or scramble siRNA and then infected with the A/Puerto Rico/8/1934 (H1N1) strain of IAV at an MOI of 0.1 for 36 h were collected, and enumerated by plaque assays. ***p < 0.001. Error bar represents standard error, which was calculated from the results of three independent sets of experiments (each dot represents the result from an experiment).
(EPS)

S3 Fig Influence of OTUB1 on IAV transcription and RNA replication. Vero-E6 cells were transfected with OTUB1 siRNA or scramble siRNA and then infected with the A/Puerto Rico/8/1934 (H1N1) strain **(A)** or A/Hong Kong/8/68 (H3N2) strain **(B)** of IAV at an MOI of 2. At 4 h, 8 h, and 12 h after infection, levels of NA mRNA, cRNA, and vRNA were determined by RT-qPCR. The amount of mRNA, cRNA, or vRNA levels of NA of A/Puerto Rico/8/34 (H1N1) or PB1 of A/Hong Kong/8/68 (H3N2) in Vero-E6 cells transfected with scramble siRNA was set as 1 at each indicated time point. Error bars represent standard error, which was calculated from the results of two independent sets of experiments.
(EPS)

S4 Fig Expression of OTUB1 and viral proteins in IAV-infected A549 cells. A549 cells were mock-infected (MOCK) **(A)**, or infected by A/Puerto Rico/8/1934 (H1N1) strain at an MOI of 2 **(B)**. At the indicated time, proteins in the lysate were determined by immunoblotting with anti-OTUB1, anti-α-tubulin, anti-PB2, anti-NP, anti-M1, anti-NS1, and anti-NS2 antibodies. The intensity of the OTUB1 bands was measured with ImageJ and normalized to the intensity of the respective α-tubulin control band. The amount of OTUB1 at Hour 0 was set to 1.
(EPS)

S1 Table Primers used for RT-qPCR. (DOCX)

S2 Table Lysine residues at amino acid positions 18, 39, 64, 72, 86, and 88 in NS2 in human IAV strains. (DOCX)

S1 Data Datasheet containing the underlying numerical data and statistical analysis for Figs 1H, 6, 7B, 8A, 8B, 9A, 9C and 9D. (XLSX)
