# Supplementary material for: OTUB1 contributes to the stability and function of Influenza A virus NS2
Source: PLoS Pathog. 2024 May 30;20(5):e1012279. doi: 10.1371/journal.ppat.1012279 (PMC11166342; doi:10.1371/journal.ppat.1012279)
Supplement: S1 Table — (DOCX) [file ppat.1012279.s005.docx]

**S1 Table. Primers used for RT-qPCR**

| A/Puerto Rico/8/34(H1N1) | mRNA | Reverse transcription | 5’-CCAGATCGTTCGAGTCGTTTTTTTTTTTTTTTTTGAACAGACTAC |
| --- | --- | --- | --- |
|  |  | qPCR | 5’-CCAGATCGTTCGAGTCGT |
|  |  |  | 5’-TGAATAGTGATACTGTAGATTGGTCT |
|  | cRNA | Reverse transcription | 5’-GCTAGCTTCAGCTAGGCATCAGTAGAAACAAGGAGTTTTTTGAAC |
|  |  | qPCR | 5’-GCTAGCTTCAGCTAGGCATC |
|  |  |  | 5’-TGAATAGTGATACTGTAGATTGGTCT |
|  | vRNA | Reverse transcription | 5’-GGCCGTCATGGTGGCGAATCCTGATACCGGCAAAGTGATGTGTGTG |
|  |  | qPCR | 5’-GGCCGTCATGGTGGCGAAT |
|  |  |  | 5’-CTCCATCAACATACACTGGACCACAGC |
| A/Hong Kong/8/68(H3N2) | mRNA | Reverse transcription | 5’-CCAGATCGTTCGAGTCGTTTTTTTTTTTTTTTTTGCCGTCTGAGC |
|  |  | qPCR | 5’-CCAGATCGTTCGAGTCGT |
|  |  |  | 5’-GGAGACCGGTTGGAATTTCC |
|  | cRNA | Reverse transcription | 5’-GCTAGCTTCAGCTAGGCATCTTTCATGAAGGACAAGCTAAATTCAC |
|  |  | qPCR | 5’-GCTAGCTTCAGCTAGGCATC |
|  |  |  | 5’-GGAGACCGGTTGGAATTTCC |
|  | vRNA | Reverse transcription | 5’-GGCCGTCATGGTGGCGAATGGCACAGCTTCATTGAGTCCTGG |
|  |  | qPCR | 5’-GGCCGTCATGGTGGCGAAT |
|  |  |  | 5’-GGATTGGAGTCCATCCCACCAGT |
