# Supplementary material for: OTUB1 contributes to the stability and function of Influenza A virus NS2
Source: PLoS Pathog. 2024 May 30;20(5):e1012279. doi: 10.1371/journal.ppat.1012279 (PMC11166342; doi:10.1371/journal.ppat.1012279)
Supplement: S2 Table — (DOCX) [file ppat.1012279.s006.docx]

**S2 Table. Lysine residues at amino acid positions 18, 39, 64, 72, 86, and 88 in NS2 in human IAV strains**

|  | **HA type** | | | | |
| --- | --- | --- | --- | --- | --- |
|  | **H1** | **H3** | **H5** | **H7** | **H9** |
| **K18** | 99.49% | 99.88% | 97.10% | 100% | 100% |
| **K39** | 98.43% | 99.45% | 98.00% | 100% | 100% |
| **K64** | 98.18% | 99.05% | 91.20% | 98% | 100% |
| **K72** | 99.49% | 99.80% | 90.20% | 100% | 100% |
| **K86** | 15.6%K/84.4% R | 99.53% R | 95.1%R | 98% R | 89% R/11% K |
| **K88** | 98.33% | 20.7% K/79.3% R | 96.10% | 97% | 100% |
| **Uni^#^** | 1974 | 2531 | 102 | 63 | 9 |
| **Sum^&^** | 17472 | 30835 | 216 | 127 | 11 |

^#^, number of human IAV strains with unique residues

^&^, number of human IAV strains analyzed in this study
